# The social epidemiology of binge-eating disorder and behaviors in early adolescents

**DOI:** 10.1186/s40337-023-00904-x

**Published:** 2023-10-13

**Authors:** Jason M. Nagata, Zacariah Smith-Russack, Angel Paul, Geomarie Ashley Saldana, Iris Y. Shao, Abubakr A. A. Al-Shoaibi, Anita V. Chaphekar, Amanda E. Downey, Jinbo He, Stuart B. Murray, Fiona C. Baker, Kyle T. Ganson

**Affiliations:** 1 Department of Pediatrics, University of California, 550 16th Street, 4th Floor, Box 0503, San Francisco, CA 94143 USA; 2 Department of Psychiatry and Behavioral Sciences, University of California, 675 18th Street, San Francisco, CA 94143 USA; 3 School of Humanities and Social Science, The Chinese University of Hong Kong, 2001 Longxiang Boulevard, Longgang District, Shenzhen, 518172 China; 4 Department of Psychiatry and Behavioral Sciences, University of Southern California, 2250 Alcazar Street, Suite 2200, Los Angeles, CA 90033 USA; 5 Center for Health Sciences, SRI International, 333 Ravenswood Ave, Menlo Park, CA 94025 USA; 6 School of Physiology, University of the Witwatersrand, 1 Jan Smuts Ave, Braamfontein, Johannesburg, 2000 South Africa; 7 Factor-Inwentash Faculty of Social Work, University of Toronto, 246 Bloor Street W, Toronto, ON M5S 1V4 Canada

**Keywords:** Eating disorders, Binge-eating disorder, Feeding and eating disorders, Adolescent, LGBTQ +, Race, Social epidemiology

## Abstract

**Background:**

Binge-eating disorder (BED) is the most common eating disorder phenotype and is linked to several negative health outcomes. Yet, little is known about the social epidemiology of BED, particularly in early adolescence. The objective of this study was to examine the associations between sociodemographic characteristics and BED and binge-eating behaviors in a large, national cohort of 10–14-year-old adolescents in the United States (U.S.)

**Methods:**

We conducted a cross-sectional analysis of two-year follow-up data from the Adolescent Brain Cognitive Development (ABCD) Study (2018 − 2020) that included 10,197 early adolescents (10 − 14 years, mean 12 years) in the U.S. Multivariable logistic regression models were used to assess the associations between sociodemographic characteristics and BED and binge-eating behaviors, defined based on the Kiddie Schedule for Affective Disorders and Schizophrenia.

**Results:**

In this early adolescent sample (48.8% female, 54.0% White, 19.8% Latino/Hispanic, 16.1% Black, 5.4% Asian, 3.2% Native American, 1.5% Other), the prevalence of BED and binge-eating behaviors were 1.0% and 6.3%, respectively. Identifying as gay or bisexual (compared to heterosexual; adjusted odds ratio [AOR]: 2.25, 95% CI 1.01–5.01) and having a household income of less than $75,000 (AOR: 2.05, 95% CI: 1.21–3.46) were associated with greater odds of BED. Being male (AOR: 1.28, 95% CI: 1.06–1.55), of Native American (AOR: 1.60, 95% CI: 1.01–2.55) descent, having a household income less than $75,000 (AOR: 1.34, 95% CI: 1.08–1.65), or identifying as gay or bisexual (AOR for ‘Yes’ Response: 1.95, 95% CI: 1.31–2.91 and AOR for ‘Maybe’ Response: 1.81, 95% CI: 1.19–2.76) were all associated with higher odds of binge-eating behaviors.

**Conclusion:**

Several sociodemographic variables showed significant associations with binge-eating behaviors, which can inform targeted screening, prevention, and education campaigns for BED among early adolescents.

**Supplementary Information:**

The online version contains supplementary material available at 10.1186/s40337-023-00904-x.

## Abstract (plain language summary)

Binge-eating disorder is the most common eating disorder, yet little research has characterized the social and demographic factors that contribute to it, especially in adolescents. We studied the social and demographic risk factors for binge-eating disorder and binge-eating behaviors for U.S. children aged 10–14. Low household income, identifying as gay or bisexual, and being of Native American descent were associated with engagement in binge-eating behaviors. Low household income and identifying as gay or bisexual were associated with binge-eating disorder. These results can help better identify, prevent, and spread awareness of binge-eating disorder.

**Supplementary Information:**

The online version contains supplementary material available at 10.1186/s40337-023-00904-x.

## Background

The most prevalent of all eating disorder phenotypes, binge-eating disorder (BED), is defined as frequently consuming unusually large amounts of food and feeling unable to stop eating. BED affects 3–5% of the US population [1–3] and acts as a precursor to an array of medical and psychiatric sequelae, including diabetes, metabolic syndrome, cardiovascular disease, and elevated suicidality [4–6]. Despite being common in the U.S. population, it has been under-studied in comparison to other eating disorders (e.g., anorexia nervosa and bulimia nervosa), and little has been written about its sociodemographic risk factors. The prevalence of BED peaks in late adolescence; however, only 11.9% of adolescents with BED seek clinical care [1, 2], underscoring the importance of early identification and prevention. Additionally, binge-eating behaviors (such as eating an amount of food that exceeds a typical amount or feeling a lack of control over one’s eating) are core features of BED [7] and are thought to be far more prevalent than the clinical diagnosis of BED and can still lead to adverse mental and physical health outcomes as well as BED later in life [1, 3]. One study found that binge-eating behaviors predicted depressive symptoms in adolescents aged 12–19 years [8]. Thus, both BED and binge-eating behaviors are important to study amongst adolescent populations.

Risk factors identified in previous studies of adolescents were mostly limited to children in larger bodies, negative comments about one’s weight, anxiety, depression, childhood adversity, food insecurity, and racism [7, 9–12]. Existing studies in adults have pointed to lower socioeconomic status [13, 14] and gay, lesbian, or bisexual sexual orientation [15, 16] as being associated with binge-eating behaviors or BED. Studies in adults have not found significant differences in binge eating by race/ethnicity [13, 14, 17]. Current literature on the social epidemiology of BED in adolescents is limited in quantity and scope, mostly focusing on a singular exposure of one sociodemographic factor. Existing studies in adolescents have pointed to lower socioeconomic status [18], identifying as gay or bisexual [19, 20], and Asian, Native American, and Hispanic race/ethnicity [21, 22] being associated with binge-eating behaviors or BED, but the association between a comprehensive sociodemographic profile and BED/binge-eating behaviors among adolescents has not been previously assessed within a large nationwide study in the U.S. or elsewhere [19–21, 23]. Much of the existing literature on eating disorders does not reflect the diverse population afflicted with eating disorder diagnoses and behaviors [24]. Critical questions regarding the social epidemiology of BED remain, including the associations of race/ethnicity, sex, sexual orientation, age, and socioeconomic status on the prevalence of BED and binge-eating behaviors.

The objective of this study was to determine the associations between sociodemographic variables and BED and binge-eating behaviors in a population-based, demographically diverse cohort of adolescents (age range: 10–14 years) participating in the two-year follow-up of the Adolescent Brain Cognitive Development (ABCD) study in the US. We aimed to determine which sociodemographic factors (including age, race/ethnicity, sex, sexual orientation, household income, and parental education) were associated with BED and binge-eating behaviors in early adolescents.

## Methods

### Study population and recruitment

We conducted a cross-sectional study using data from the Adolescent Brain Cognitive Development (ABCD) Study. The ABCD study is a multi-center cohort study designed to assess brain development and adolescent health across 11,875 children recruited from 21 geographically diverse sites around the United States. Participants were mostly recruited and sampled through schools, taking into consideration gender, race/ethnicity, socioeconomic status, and urbanicity to minimize sample selection bias [25]. More specifically, the ABCD Study employed probability sampling of US schools within the 21 catchment areas as the primary method to identify which schools to target for recruitment. The 21 catchment areas were geographically distributed to the nation’s four major regions (Northeast, South, Midwest, West) and were demographically and socioeconomically diverse. The ABCD Study used annual databases maintained by the National Center for Education Statistics, which provided sociodemographic characteristics of students attending each public and private school in the catchment areas of the 21 study sites. The ABCD Study selected a stratified, probability sample of schools from the sampling frame for each of the 21 sites, minimizing systematic sampling biases in recruitment at the school level. Thus, the sample is epidemiologically informed; however, it may not be fully representative of the US population since research participation was voluntary, based on self-selection. Incompleteness at any particular school and the response rates for individual students within the schools are not incorporated into the sample weighting schema [26]. Further details of the ABCD Study design and recruitment have been previously described [25].

At baseline (2016–2018, ages 9–10 years), the prevalence of BED in the cohort was only 0.7% [27]; therefore, sociodemographic analyses may have been underpowered. Data analyzed here are therefore from the ABCD 4.0 release for the two-year follow-up (2018–2020), when participants ranged in age between 10 and 14 years, with most being 11–12 years old. There were missing data for BED or binge-eating behaviors at the two-year follow-up (*n* = 1,678) or missing sociodemographic data (*n* = 1,016). Comparisons of participants with missing versus not missing binge-eating data are shown in Additional File 1. Centralized institutional review board (IRB) approval was obtained from the University of California, San Diego. Study sites obtained approval from their local IRBs. Caregivers provided written informed consent, and each child provided written assent.

### Independent variables: sociodemographic characteristics

Sex at birth was defined as male or female. Annual household income was defined as a binary variable based on being above or below the median income cutoff of $75,000 per year (approximately the median annual household income in the U.S. during the study period). Parent education was a binary variable that differentiated “high school or less” and “college or more.” Race/ethnicity was categorical, with options being White, Latino/Hispanic, Black, Asian, Native American, and Other. Sex, household income, parental education, and race were based on parents’ self-report and were collected during the baseline visit of the ABCD study. ABCD Study site identification was included as a confounder to adjust for potential regional variation, given regional variation in sociodemographic factors as well as eating disorder prevalence in the U.S. [28]. Children were asked whether they were gay or bisexual during their baseline surveys, and their response was the sexual orientation variable.

### Outcome: BED and binge-eating behaviors

BED was assessed at the two-year follow-up via the Kiddie Schedule for Affective Disorders and Schizophrenia (KSADS-5), an online tool for categorizing child and adolescent mental health concerns based on the DSM-5 definitions [12, 29, 30]. Parents/caregivers completed the survey on the frequency, duration, and traits of their child’s binge-eating behaviors. BED was defined based on responses to the interview questions that corresponded to the DSM-5 criteria for BED using the KSADS-5 computerized scoring system (Additional File 2). Binge-eating behaviors were defined as the reporting of any episode of eating more than intended and loss of control while eating in the past two weeks. Specific validation data for BED in KSADS-5 have not been reported, although the approach was similar for other DSM-5 diagnoses (e.g., depressive disorders, anxiety disorders) which have good validation data [30]. In general, the KSADS has demonstrated good convergent validity with clinical rating scales [30], and there is good to excellent concordance between parent and adolescent self-administered KSADS data [30]. Although prior studies have shown low concordance among parent and child reports of binge eating [31, 32], parents are important reporters for eating disorders in children given children have less insight regarding their eating behaviors [33, 34].

### Statistical analyses

To assess the associations between sociodemographic characteristics and BED and binge-eating behaviors, we used multivariable logistic regression models. The adjusted models estimated the association between sociodemographic variables of interest (race/ethnicity, sex, sexual orientation, household income, and parent education), and BED and binge-eating behaviors and adjusted for study site. Analyses were conducted using Stata 18.0 (StataCorp, College Station, TX). The ABCD study’s sociodemographic variables were standardized using propensity score weighting to match the distribution of the nationally representative American Community Survey from the U.S. Census [35]. To address missing data for sociodemographic variables, we applied multiple imputation with chained equations, with 10 imputed datasets formed.

## Results

The study sample consisted of 10,197 individuals with a mean age of 12 years (51.2% male, 48.8% female, Table 1). There was a 1.0% prevalence of BED and 6.3% prevalence of binge-eating behaviors within the study population.

**Table 1:** Sociodemographic and binge eating characteristics of Adolescent Brain Cognitive Development (ABCD) Study participants (N = 10,197)

Sociodemographic characteristics	Totals	Binge-eating disorder			Binge-eating behavior		
		Yes	No	P-Value	Yes	No	P-Value
Age (years)	12.03	12.03	12.00	0.562	12.06	12	0.103
Sex (%)	–	–	–	0.869	–	–	0.087
Female	48.8%	51.4%	48.8%		45.0%	49.1%	
Male	51.2%	48.6%	51.2%		55.0%	50.9%	
Race/ethnicity (%)	–	–	–	0.073	–	–	< 0.001
White	54.0%	54.0%	52.1%		45.8%	54.6%	
Latino / Hispanic	19.8%	20.4%	19.8%		26.7%	19.4%	
Black	16.1%	21.7%	16.0%		14.7%	16.2%	
Asian	5.4%	0.0%	5.5%		6.4%	5.3%	
Native American	3.2%	3.2%	5.1%		5.3%	3.0%	
Other	1.5%	0.8%	1.5%		1.2%	1.5%	
Household income (%)	–	–	–	0.001	–	–	< 0.001
Less than or equal to $75,000	54.9%	74.2%	54.7%		65.1%	54.2%	
More than $75,000	45.1%	45.3%	25.8%		34.9%	45.8%	
Parents' highest education (%)	–	–	–	0.376	–	–	0.013
High school education or less	18.4%	21.6%	18.4%		22.5%	18.2%	
College education or more	81.6%	78.4%	81.6%		77.5%	81.8%	
Gay or bisexual (%)	–	–	–	< 0.001	–	–	0.008
No	87.3%	79.1%	87.4%		82.5%	87.7%	
Yes	4.4%	9.8%	4.3%		7.1%	4.2%	
Maybe	3.8%	6.0%	3.8%		5.6%	3.7%	
Don't understand the question	3.2%	0.8%	3.2%		3.5%	3.2%	

ABCD propensity weights were applied to yield estimates based on the American Community Survey from the US Census

Table 2 shows unadjusted and adjusted sociodemographic associations with BED versus no BED using logistic regression. Participants with a household income of less than $75,000 had two times the odds of developing BED than those with a household income over $75,000 (adjusted odds ratio [AOR]: 2.05, 95% confidence interval [CI]: 1.21–3.46). Identifying as gay or bisexual (AOR: 2.25, 95% CI: 1.01–5.01) was associated with BED compared to their heterosexual peers.

**Table 2:** Unadjusted and adjusted logistic regression analysis of sociodemographic associations with binge-eating disorder in the Adolescent Brain Cognitive Development (ABCD) Study

	Binge-eating disorder		Binge-eating disorder	
Sociodemographic characteristics	Unadjusted odds ratio (95% CI)	*p*	Adjusted odds ratio (95% CI)	*p*
Age (years)	1.11 (0.77, 1.59)	0.56	1.15 (0.80, 1.67)	0.44
Sex	–	–	–	–
Female	Reference	Reference	Reference	Reference
Male	0.90 (0.58, 1.39)	0.64	1.03 0.64, 1.62)	0.91
Race/ethnicity	–	–	–	–
White	Reference	Reference	Reference	Reference
Latino / Hispanic	1.06 (0.59, 1.89)	0.83	0.74 (0.34, 1.65)	0.80
Black	1.40 (0.81, 2.39)	0.22	1.08 (0.55, 2.11)	0.82
Asian	–	–	–	–
Native American	1.66 (0.57, 4.75)	0.35	1.01 (0.31, 3.38)	0.98
Other	0.57 (0.07, 4.21)	0.58	0.52 (0.07, 3.93)	0.53
Household income	–	–	–	–
Less than or equal to $75,000	**2.25 (1.45, 3.49)**	** < 0.001**	**2.05 (1.21, 3.46)**	**0.008**
More than $75,000	Reference	Reference	Reference	Reference
Parents' highest education	–	–	–	–
High school education or less	0.82 (0.48, 1.41)	0.47	1.11 (0.61, 2.02)	0.73
College education or more	Reference	Reference	Reference	Reference
Gay or bisexual	–	–	–	–
No	Reference	Reference	Reference	Reference
Yes	**2.50 (1.17, 5.30)**	**0.017**	**2.25 (1.01, 5.01)**	**0.048**
Maybe	1.77 (0.68, 4.55)	0.236	1.73 (0.66, 4.50)	0.264
Don't understand the question	0.29 (0.04, 2.07)	0.22	0.30 (0.04, 2.21)	0.26

Bold indicates *p* < 0.05. Adjusted models include adjustment for age, sex, race/ethnicity, sexual orientation, household income, parent education, and study site. Propensity weights from the Adolescent Brain Cognitive Development Study were applied based on the American Community Survey from the US Census

A similar logistic regression model examined the associations between sociodemographic characteristics and binge-eating behavior versus no binge-eating behavior (Table 3). Each additional year of age was associated with higher odds (AOR: 1.19, 95% CI 1.04 – 1.37) of binge-eating behaviors. Male adolescents had higher odds of binge-eating behaviors than their female counterparts (AOR: 1.28, 95% CI 1.06–1.55). Additionally, participants from a household with an income less than $75,000 had higher odds of binge-eating behaviors (AOR = 1.34, 95% CI 1.08–1.65). Individuals of Native American (AOR: 1.60, 95% CI: 1.01–2.55) descent had higher odds ratios of binge-eating behaviors compared to White adolescents. Significant associations were also found for adolescents who answered “Yes” (AOR: 1.95, 95% CI: 1.31–2.91) and “Maybe” (AOR: 1.81, 95% CI: 1.19, 2.76) to identifying as gay or bisexual (compared to “No”) and binge-eating behaviors.

**Table 3:** Unadjusted and adjusted logistic regression analysis of sociodemographic associations with binge-eating behaviors in the Adolescent Brain Cognitive Development (ABCD) Study

	Binge-eating behavior		Binge-eating behavior	
Sociodemographic characteristics	Unadjusted odds ratio (95% CI)	*p*	Adjusted odds ratio (95% CI)	*p*
Age (years)	1.12 (0.98, 1.29)	0.10	**1.19 (1.04, 1.37)**	**0.01**
Sex	–	–	–	–
Female	Reference	Reference	Reference	Reference
Male	1.18 (0.98, 1.41)	0.08	**1.28 (1.06, 1.55)**	**0.01**
Race/ethnicity	–	–	–	–
White	Reference	Reference	Reference	Reference
Latino / Hispanic	**1.64 (1.31, 2.07)**	** < 0.001**	1.29 (0.96, 1.73)	0.09
Black	1.09 (0.87, 1.39)	0.44	0.99 (0.75, 1.31)	0.97
Asian	1.41 (0.91, 2.19)	0.12	1.37 (0.85, 2.21)	0.20
Native American	**2.06 (1.32, 3.22)**	**0.001**	**1.60 (1.01, 2.55)**	**0.05**
Other	0.93 (0.35, 2.42)	0.88	0.92 (0.34, 2.43)	0.86
Household income	–	–	–	–
Less than or equal to $75,000	**1.54 (1.28, 1.83)**	** < 0.001**	**1.34 (1.08, 1.65)**	**0.007**
More than $75,000	Reference	Reference	Reference	Reference
Parents' highest education	–	–	–	–
High school education or less	**0.76 (0.60, 0.95)**	**0.02**	0.94 (0.72, 1.21)	0.64
College education or more	Reference	Reference	Reference	Reference
Gay or bisexual	–	–	–	–
No	Reference	Reference	Reference	Reference
Yes	**1.79 (1.21, 2.64)**	**0.003**	**1.95 (1.31, 2.91)**	**0.001**
Maybe	**1.62 (1.07, 2.45)**	**0.02**	**1.81 (1.19, 2.76)**	**0.005**
Don't understand the question	1.17 (0.72, 1.91)	0.52	1.19 (0.72, 1.95)	0.48

Bold indicates *p* < 0.05. Adjusted models include adjustment for age, sex, race/ethnicity, sexual orientation, household income, parent education, and study site. Propensity weights from the Adolescent Brain Cognitive Development Study were applied based on the American Community Survey from the US Census

## Discussion

In this demographically diverse national sample of 10–14-year-old early adolescents in the US, we found a 1.0% prevalence of BED and 6.3% prevalence of binge-eating behaviors, which is similar to that reported in prior research, with approximately 1.3% of children and adolescents meeting the diagnostic criteria for BED [36]. The results also highlighted several noteworthy sociodemographic associations with binge-eating behaviors. Lower household income was associated with both BED and binge-eating behaviors. Male sex, Native American race/ethnicity, and responding “Yes” or “Maybe” to identifying as gay or bisexual were also associated with binge-eating behaviors.

Adolescent males had higher odds of binge-eating behaviors when compared to adolescent females, highlighting the need to move away from the stereotype of eating disorders as a female disorder. In male adults and adolescents, body dissatisfaction is often tied to a drive for muscularity and larger size as opposed to thinness [37]. Over half of young men who report weight gain and bulking goals report eating more to achieve this goal [38], which leads to the consumption of larger volumes of food [39]. Consumption of larger amounts of food than what most people would eat in a discrete period of time is part of the first criterion for binge-eating disorder [29]. In the context of bulking and muscularity-oriented goals, men are more likely than women to engage in “cheat meals,” eating episodes that temporarily depart from one’s established dietary practices (e.g., restriction or restraint) to consume prohibited foods momentarily, then returning back to the previous dietary practice [40, 41]. Cheat meals have been linked to overeating, loss of control while eating, and binge-eating behaviors as they can involve consuming large volumes of food (e.g., 1,000–9,000 cal) commensurate with an objective binge episode [40, 42]. Cheat meals are most often spontaneous or unplanned, which can be consistent with a loss of control [41]. A narrow, female-specific lens on disordered eating behaviors will continue to make male adolescents, with unique eating disorder (ED) presentations and behaviors, underrecognized and undertreated [43–45]. Our study found a significant association between male sex and binge-eating behaviors but not BED. Prior studies also found a low prevalence of BED in adolescent males when applying the full DSM-5 criteria but a higher prevalence when partial BED criteria are utilized [46]. The findings from our analysis further illustrate the prevalence of binge-eating behaviors in adolescent males and serve as a call for more studies focusing on eating disorders in this population, particularly on the relationship between muscularity-oriented eating goals and binge eating.

Apart from biological sex, we also examined the association between sexual orientation and the two outcomes, BED and binge-eating behaviors. Individuals who identified as gay or bisexual were found to have greater odds of BED and binge-eating behaviors, compared to their heterosexual peers. This finding is consistent with previous studies showing that those who identify as gay or bisexual have greater odds of disordered eating [20]. Adolescents who identify as gay and bisexual face external and internal stressors, such as stigma, bullying, discrimination, and internalized homophobia, which all compound to an increased risk for disordered eating [47]. Our findings aligned with a 2018 study examining sexual orientation and disordered eating symptoms among 16-year-old adolescents in the UK [19]. This study found that adolescent males who identified as gay or bisexual had 12.5 times the odds of binge-eating compared to their heterosexual counterparts [18]. Similarly, adolescent girls who identified as lesbian or bisexual had twice the odds of binge eating and purging compared to their heterosexual counterparts [18]. We also found a significant association between those who responded ‘maybe’ and binge-eating behaviors. Given the emerging research that supports this association, future studies should explore the prevention, early identification, and management strategies of binge-eating behaviors for gay or bisexual adolescents.

Our study found that adolescents of Native American descent had higher odds of binge-eating behaviors compared to their White counterparts. Previous studies have reported a higher prevalence of disordered eating, including binge-eating, in Native American populations compared to White groups [21]. A higher prevalence is seen when a broader definition of binge-eating disorder is applied, but when stricter DSM criteria are set, this number decreases [48]. Cultural stigma is a potential source of under-reporting in Native American groups, and future studies must be conducted on the effectiveness of culturally sensitive educational interventions surrounding binge-eating behaviors and BED. More culturally sensitive approaches in existing and new policies/programs relating to binge-eating behaviors and BED are likely to support those adolescents who may be underrecognized because of cultural stigma, shame, or not meeting full BED diagnostic criteria.

We also found that individuals from households with lower annual incomes (< $75,000) were significantly associated with both BED and binge-eating behaviors. This finding further supports the growing body of literature, which stands in contrast to the myth that eating disorders are a “disease of affluence” [49]. One particular 2018 study found a 15% prevalence of self-reported eating disorders in low socioeconomic status (SES) participants, higher than the 3% U.S. national average, further directing the notion that those in low-income settings have a significant prevalence of eating disorders [18]. Food insecurity has been shown to have an association with binge-eating among both adolescents [12] and parents [50]. Our findings highlight the need for policies that address food insecurity and poverty as risk factors for disordered eating, as well as targeted efforts to support those with lower SES who may struggle with binge eating.

Several potential limitations should be noted. The prevalence of BED was relatively low; however, prevalence is expected to rise as the cohort ages. Additionally, because of the observational nature of the analysis, we cannot make claims beyond speculation about the directionality of our associations. Because many of the sociodemographic variables and questions on the KSADS-5 questionnaire are self-reported by parents and adolescents, the estimates are subject to reporting bias. Selection bias is possible as participants excluded due to missing outcome data were more likely to have lower household income, lower parent education, and identify as Black, Latino/Hispanic, or Asian (Additional File 1). Despite these limitations, the strengths of the study include a large, diverse, population-based sample focusing on BED and binge-eating behaviors in early adolescence.

The results highlight the need for further investigation into adolescent screening for eating disorders, specifically those involving binge-eating behaviors. As per the US Preventive Services Task Force (USPSTF), there is currently inadequate evidence to recommend universal eating disorder screening in adolescents [51]. These findings illustrate the extent to which eating disorders present differently in diverse populations, thus contributing to underdiagnosis and under recognition of disordered eating. Males, sexual minorities, and low-income populations require targeted screening and intervention strategies to support those who may be at risk for disordered eating. Given the barriers to care that many people with BED face, clinicians must better understand culturally sensitive strategies for screening, identification, and management of this disorder.

### Supplementary Information

**Additional file 1:** Comparison of participants included versus excluded.

**Additional file 2:** Kiddie Schedule for Affective Disorders and Schizophrenia (KSADS-5) assessment of binge-eating disorder (BEDa) and binge-eating behaviorb in the ABCD Study. 

## Abbreviations

ABCD: Adolescent brain cognitive development study
ACEs: Adverse childhood experiences
EDs: eating disorders
BED: binge-eating disorder
CDC: Centers for disease control and prevention
PTSD: post-traumatic stress disorder
IRB: institutional review board
KSADS-5: Kiddie schedule for affective disorders and schizophrenia

## Declarations

### Availability of data and materials

Data used in the preparation of this article were obtained from the ABCD Study (https://abcdstudy.org), held in the NIMH Data Archive (NDA).
