# Supplementary material for: The social epidemiology of binge-eating disorder and behaviors in early adolescents
Source: J Eat Disord. 2023 Oct 13;11:182. doi: 10.1186/s40337-023-00904-x (PMC10571438; doi:10.1186/s40337-023-00904-x)
Supplement: Supplementary file 1 — Additional file 1: Comparison of participants included versus excluded. [file 40337_2023_904_MOESM1_ESM.docx]

| Supplemental File 1. Comparison of participants included versus excluded | | | |  |
| --- | --- | --- | --- | --- |
| Sociodemographic characteristics | Included (n=10,197) | Excluded (n=1,678) | p | |
| Age (years) | 12.03 | 12.28 | <0.001 | |
| Sex (%) | --------- | --------- | 0.932 | |
| Female | 48.8% | 49.0% |  | |
| Male | 51.2% | 51.0% |  | |
| Race/ethnicity (%) | --------- | --------- | <0.001 | |
| White | 54.0% | 42.8% |  | |
| Latino / Hispanic | 19.8% | 21.7% |  | |
| Black | 16.1% | 24.9% |  | |
| Asian | 5.4% | 6.1% |  | |
| Native American | 3.2% | 3.1% |  | |
| Other | 1.5% | 1.4% |  | |
| Household income (%) | --------- | --------- | <0.001 | |
| Less than or equal to $75,000 | 54.9% | 66.7% |  | |
| More than $75,000 | 45.1% | 33.3% |  | |
| Parents' highest education (%) | --------- | --------- | <0.001 | |
| High school education or less | 18.4% | 31.1% |  | |
| College education or more | 81.6% | 68.9% |  | |
| Sexual Minority Status | --------- | --------- | 0.478 | |
| No | 87.3% | 91.0% |  | |
| Yes | 4.4% | 4.3% |  | |
| Maybe | 3.8% | 2.7% |  | |
| Don't understand the question | 3.2% | 1.0% |  | |
