# Supplementary material for: The social epidemiology of binge-eating disorder and behaviors in early adolescents
Source: J Eat Disord. 2023 Oct 13;11:182. doi: 10.1186/s40337-023-00904-x (PMC10571438; doi:10.1186/s40337-023-00904-x)
Supplement: Supplementary file 2 — Additional file 2: Kiddie Schedule for Affective Disorders and Schizophrenia (KSADS-5) assessment of binge-eating disorder (BEDa) and binge-eating behaviorb in the ABCD Study. [file 40337_2023_904_MOESM2_ESM.docx]

| Supplemental File 2. Kiddie Schedule for Affective Disorders and Schizophrenia (KSADS-5) assessment of binge-eating disorder (BED^a^) and binge-eating behavior^b^ in the ABCD Study | | |
| --- | --- | --- |
| **Diagnostic Criteria** | **Question ID** | **Question** |
| Binge-eating behavior^a^ | 1.13.5.Q1 | In the past two weeks, how often has your child had eating binges, when he or she lost control of their eating and ate way more than he or she needed to because your child was unable to stop himself or herself from eating? |
| Frequency of binge eating | 2.13.9.Q1a | You said before that sometimes your child binge eats. Over average, does he or she binge eat at least once a week? |
|  | 2.13.9.Q1b | How long has your child been binge eating at least once a week? Please entire weeks, months, or years. |
| Characteristics of binge eating | 2.13.10.Q1 | Note below all the things that are true when your child binge eats |
|  | 2.13.10.Q1a | My child eats much more rapidly than normal |
|  | 2.13.10.Q1b | My child eats a lot even though he or she is not hungry |
|  | 2.13.10.Q1c | My child eats alone because he or she is embarrassed |
|  | 2.13.10.Q1c | My child feels disgusted or guilty after binge eating |
|  | 2.13.10.Q1d | My child feels depressed after binge eating |
|  | 2.13.10.Q1e | My child feels like he or she has no control when they binge eat |
| Distress associated with binge eating | 2.13.11.Q1b | How much discomfort or distress does binge eating cause your child? |
| ^a^BED was based on the following criteria: binge eating present, frequency of binge-eating at least once a week for at least 3 months (or 12 weeks), at least three characteristics of binge-eating, distress associated with binge-eating present, no current compensatory behavior and does not meet criteria for current diagnosis of bulimia nervosa or anorexia nervosa | | |
| ^b^Binge-eating behavior was based on the binge-eating behavior question. Any binge-eating behavior (frequency of one or more in the past two weeks) was coded as an affirmative for binge-eating behavior. | | |
